# Key role of mitochondrial mutation Leu107Ser (COX1) in deltamethrin resistance in salmon lice (*Lepeophtheirus salmonis*)

**DOI:** 10.1038/s41598-022-14023-1

**Published:** 2022-06-20

**Authors:** Claudia Tschesche, Michaël Bekaert, David I. Bassett, Sally Boyd, James E. Bron, Armin Sturm

**Affiliations:** Institute of Aquaculture, Faculty of Natural Sciences, University of Stirling, Stirling, FK9 4LA UK

**Keywords:** Evolution, Genetics, Ocean sciences

## Abstract

The pyrethroid deltamethrin (DTM) is used to treat Atlantic salmon (*Salmo salar*) against salmon louse (*Lepeophtheirus salmonis*) infestations. However, DTM resistance has evolved in *L. salmonis* and is currently common in the North Atlantic. This study aimed to re-assess the association between DTM resistance and mitochondrial (mtDNA) mutations demonstrated in previous reports. Among 218 *L. salmonis* collected in Scotland in 2018–2019, 89.4% showed DTM resistance in bioassays, while 93.6% expressed at least one of four mtDNA single nucleotide polymorphisms (SNPs) previously shown to be resistance associated. Genotyping at further 14 SNP loci allowed to define three resistance-associated mtDNA haplotypes, named 2, 3 and 4, occurring in 72.0%, 14.2% and 7.3% of samples, respectively. *L. salmonis* strains IoA-02 (haplotype 2) and IoA-10 (haplotype 3) both showed high levels (~ 100-fold) of DTM resistance, which was inherited maternally in crossing experiments. MtDNA haplotypes 2 and 3 differed in genotype for 17 of 18 studied SNPs, but shared one mutation that causes an amino acid change (Leu107Ser) in the cytochrome c oxidase subunit 1 (COX1) and was present in all DTM resistant while lacking in all susceptible parasites. We conclude that Leu107Ser (COX1) is a main genetic determinant of DTM resistance in *L. salmonis.*

## Introduction

Caligid sea lice (Copepoda: Crustacea) are ectoparasites of wild and farmed marine fish, which feed on the mucus, skin and blood of their hosts^1^. In the most severe cases, infestations can lead to skin lesions, secondary infections, anaemia, osmoregulatory imbalances, induction of endocrine stress responses, reduced appetite and growth, immunosuppression and, if untreated, potentially death^2,3^. Sea lice infections are a major health management challenge for the commercial mariculture of Atlantic salmon (*Salmo salar* L.). The salmon louse *Lepeophtheirus salmonis* (Krøyer, 1837) is the main caligid affecting salmon farming in the northern hemisphere^4^, with two subspecies being distinguished that differ in geographic distribution and range of wild host species^5^. *L. salmonis salmonis* is found in the Northern Atlantic and occurs on wild populations of Atlantic salmon, sea trout (*Salmo trutta* L.) and arctic char (*Salvelinus alpinus* L.), while *L. salmonis oncorhynchi* occurs in the Northern Pacific where wild hosts include Pacific salmon of the genus *Oncorhynchus*. In the southern hemisphere, salmon aquaculture is impacted by the sea louse *Caligus rogercresseyi*, the natural hosts of which are different non-salmonid fish species^6^. In 2018, the estimated global costs of sea lice infections to the salmon farming industry were approximately US $873 million/£700 million^7^, comprising mainly treatment costs and to a lesser extent losses in production.

Sea lice infections of farmed salmon are controlled by integrated pest management strategies combining farm management measures, such as fallowing and the use of single year classes, with a diverse array of non-medicinal control approaches and a limited range of licensed veterinary drugs^4,8^. Non-medicinal sea lice control approaches include alternative cage designs reducing infection pressure^9^, different systems of salmon delousing using physical means such as the application of water jets or brief immersion in warm water^10^, and the co-culture of Atlantic salmon with different species of cleaner fish that remove sea lice from infected salmon^11^. Veterinary drugs available for salmon delousing include the macrocyclic lactone emamectin benzoate and different benzoylureas, which are applied as feed additives, and the organophosphate azamethiphos, the disinfectant hydrogen peroxide, and the pyrethroids cypermethrin and deltamethrin (DTM), which are supplied as bath treatments^8^.

The use of a limited range of salmon delousing agents over more than two decades has led to the evolution of drug resistance in *L. salmonis* populations of the North Atlantic, with most current veterinary treatments being affected by losses of efficacy^12,13^. Pyrethroids have been used for sea louse control in Scotland and Norway since the late 1990s. First evidence for pyrethroid resistance in *L. salmonis* in Norway, Ireland and Scotland emerged between 2001 and 2003^14^. By 2009, DTM resistance had reached high levels in Norway, with the median effective concentration (EC_50_) determined in *L. salmonis* bioassays being increased by more than 100-fold compared to reference values for susceptible parasites^12^. In Canada, DTM has been employed for salmon delousing briefly from 2009 to 2010^13^, and in Chile pyrethroids have been deployed for sea lice control since 2007^15^. In Chile, bioassay results provide evidence for DTM resistance in *C. rogercresseyi* in salmon farming areas^15^.

The toxicity of pyrethroids is based on their blocking of arthropod voltage-gated sodium channels (Na_v_), which have essential roles in neurotransmission^16^. Pyrethroid resistance in insects can be based on missense mutations of Na_v_ that decrease the channel’s affinity to the pesticides^17,18^, or result from the constitutive up-regulation of key enzymes of pesticide detoxification, such as the carboxylesterases (CaEs), cytochrome P450s (CYPs), or glutathione-S-transferases^19^. In *L. salmonis,* the molecular mechanisms of pyrethroid resistance remain to be resolved. DTM resistance in *L. salmonis* shows a predominantly maternal mode of inheritance, and is associated with single nucleotide polymorphisms (SNPs) in the mitochondrial genome (mtDNA)^20–22^. These findings suggest a novel, still unresolved resistance mechanism and imply still unidentified mitochondrial targets for DTM toxicity in susceptible lice. In support of this hypothesis, DTM has been shown to disrupt mitochondrial ATP production^21^ and induce apoptosis in skeletal muscle cells, which have a high content of mitochondria^22^. Results from crossing experiments suggested additional minor roles of unidentified nuclear genetic factors in DTM susceptibility^21^. *L. salmonis* possesses three Na_v_ genes^23^, of which LsNa_v_1.3 harbours a SNP that is homologous to a missense mutation of insect Na_v_ decreasing pyrethroid affinity^24^. However, while DTM resistance of *L. salmonis* field populations determined in acute bioassays was associated with genotypes at two mtDNA SNP loci, the SNP in LsNa_v_1.3 showed no such association and did not explain mt-DNA independent DTM resistance observed in a crossing experiment^25^. Similarly, genome-wide characterisation and transcript expression studies of *L.* *salmonis* CYP and CaE genes failed to provide evidence for an upregulation of CYPs or CaEs in DTM resistant parasites^26,27^.

While the studies reviewed above demonstrate that the mtDNA contains major genetic determinants of DTM resistance in *L. salmonis*, it is at present unclear which mtDNA mutations are involved in the resistance mechanism. Two available studies have identified 28 and 175 candidate mtDNA SNPs potentially associated with DTM resistance, respectively^21,22^. The assessment of mtDNA markers is complicated by the uniparental mode of inheritance and lack of recombination of mtDNA, which leads to mtDNA SNPs conferring a trait of interest to be transmitted to the next generation together with functionally irrelevant hitchhiking mutations. In a study of DTM resistant *L. salmonis* isolates from Scotland*,* strains originating from geographically distant farm sites showed virtually identical mtDNA sequences, suggesting the clonal expansion of one DTM-resistance-associated mtDNA haplotype as a result of drug selection^21^. However, due to the limited number of individuals analysed, the study may have failed to detect alternative rare mtDNA haplotypes associated to DTM resistance. Assessing a range of mtDNA SNPs in a larger sample of field parasites for association with DTM resistance could help differentiating functionally relevant from irrelevant mutations.

The aim of the present study was to re-assess the association of mtDNA SNPs with DTM resistance in *L. salmonis* demonstrated by earlier studies^20–22^. *L. salmonis* obtained from Scottish farm sites in 2018 and 2019 were phenotyped by DTM bioassays and genotyped at a range of mtDNA SNP loci and putatively resistance-associated mtDNA haplotypes established. A DTM resistant *L. salmonis* strain carrying a mtDNA haplotype differing from that present in a previously characterised DTM resistant strain^21^ was established and subjected to toxicological and genetic analyses. Moreover, to address the evolutionary origin of mtDNA haplotypes associated with DTM resistance, archived samples of *L. salmonis* strains established in 2011–2012 and lice collected from wild salmon in 2010 were genotyped at mtDNA SNP loci. In addition, sequences of four mtDNA genes for geographically diverse *L.* *salmonis salmonis* populations reported in an earlier study^28^ were re-analysed to compare expression of mtDNA SNP alleles to those found in this study. Based on the most parsimonious assumption that the mtDNA determinants of DTM resistance in *L. salmonis* are the same across isolates, susceptible and DTM resistance-associated mtDNA haplotypes were compared, allowing to narrow down the number of candidate mitochondrial genetic determinants of DTM resistance in *L. salmonis.*

## Materials and methods

### Ethics statement

All research projects involving the University of Stirling are subject to a thorough Ethical Review Process prior to any work being approved. The present research was assessed by the University of Stirling Animal Welfare Ethical Review Body (AWERB) and passed the ethical review process. Laboratory infections of Atlantic salmon with *L. salmonis* were performed under a valid UK Home Office license and at low parasite densities unlikely to compromise fish welfare.

### *Lepeophtheirus salmonis* strains and husbandry

In addition to salmon lice sampled at field sites (see below), different *L. salmonis* strains maintained in laboratory culture were studied, with an overview of strains provided in Supplementary Table S1. Strain IoA-00 was derived from an isolate collected in the Firth of Clyde system in 2003 and is susceptible to DTM, emamectin benzoate and azamethiphos^23,29^. Strain IoA-01, which originates from Sutherland and was taken into culture in 2008, is resistant to emamectin benzoate but susceptible to DTM^21,29^. Strain IoA-02 was established in 2011 from material collected in the Shetland islands and is resistant to emamectin benzoate, DTM, and azamethiphos^21,26,29^. IoA-03 (Sutherland, 2014), NA01-O and NA01-P (Argyll and Bute, 2012) are further DTM resistant strains^21^. DTM susceptibility and mtDNA SNP genotypes of the above strains have been characterised in an earlier report^21^. While different definitions of DTM resistance in *L. salmonis* based on bioassays results have been proposed in the literature^30,31^, strains referred to as DTM resistant in this report are characterised by DTM median effective concentration (EC_50_) values obtained in traditional bioassays (see below for definition) greater than 2 µg/L DTM, which is the level typically used in bath treatments^30^. This rather stringent definition of DTM resistance was chosen to select isolates for study which showed susceptibility phenotypes differing markedly from those found in *L. salmonis* prior to selection^14^, and is not meant to imply lower levels of DTM hyposensitivity are not clinically relevant. During experiments of this study, the well-characterised strains IoA-00 and IoA-02, which are still maintained in laboratory culture, were included to provide *L. salmonis* of known DTM susceptibility status. In addition, archived specimens of the other strains, which had been conserved in absolute ethanol followed by storage at − 20 °C, were used in genetic analyses. Finally, experiments described in this report included DTM-resistant strain IoA-10, which was established during the current study. Strains were cultured at the Marine Environmental Research Laboratory of the University of Stirling (Machrihanish, UK), with husbandry conditions described in detail elsewhere^32^.

### Collection of *L. salmonis* from field sites

Preadult-II and adult *L. salmonis* were collected at farm site 1 (Argyll and Bute) during routine veterinary procedures in November 2018. Lice were put into polyethylene bags containing cool (~ 10 °C) oxygen-saturated seawater, which were placed in insulated boxes equipped with cold packs and immediately shipped to the University of Stirling, where recipients containing lice were transferred to a temperature-controlled incubator set to 12 °C and linked to airlines. The next day, the DTM resistance phenotype of lice was characterised using bioassays (see below), after which parasites were conserved in absolute ethanol for later genotyping. In addition, genetic analyses of this study included ethanol-conserved lice collected at further sites (2: Argyll and Bute, November 2018; 3: Argyll and Bute, May 2019; 4: Sutherland, September 2019), for which DTM bioassay data have been reported previously^25^. Further parasites studied included adult *L. salmonis* collected in 2010 from wild Atlantic salmon caught in the river Esk, located on the East coast of Scotland^21^. No bioassay data were available for these lice.

To obtain *L.* *salmonis* for copepodid bioassays, gravid lice with paired egg strings were collected from farm sites 5 (Argyll and Bute), 6 and 7 (both Inverness-shire) in December 2019, followed by shipment to the University of Stirling and transfer of lice to a temperature-controlled incubator, as described above. To obtain batches of copepodids derived from the same dam, paired egg strings of individual females were removed and incubated in beakers containing seawater and linked to airlines in a temperature-controlled chamber set to 12 °C, allowing hatching of eggs and progression of larval development to the copepodid stage.

### Establishment of DTM resistant *L. salmonis* strain IoA-10

Gravid female *L. salmonis* (N = 30) were obtained from an aquaculture site located on the West coast of Scotland (Inverness-shire) in November 2019, and transported to the Marine Environmental Laboratory of the University of Stirling in Machrihanish, with details as given above. Paired egg strings were removed from dams and incubated, as described above, to produce batches of copepodids derived from the same mother. The corresponding dams were preserved in absolute ethanol, and subjected to DNA extraction and genotyping assays (see below) to establish mtDNA haplotypes. Copepodids derived from one dam showing the haplotype of interest (haplotype 3, see “Results” section) were selected and used to infect naïve host fish to establish strain IoA-10 after the mtDNA haplotype of the larval batch had been confirmed by repeating genotyping assays with a subset of copepodids.

### Crossing experiment

To obtain insights into the mode of inheritance of DTM resistance expressed by strain IoA-10, a crossing experiment between this strain and the DTM susceptible strain IoA-00 was performed in July 2020, spanning two generations termed parental (P0) and first filial generation (F1). In addition to crosses, the experiment included treatments to produce IoA-00, IoA-02 and IoA-10 lice synchronised to hybrids generated in the cross. Experimental infections for each of the above strains were performed with Atlantic salmon smolts (IoA-00 and IoA-10: 14 host fish each; IoA-02: 7 host fish). Lice were maintained on fish until development had reached the adult male and preadult-II female stages, but lice had not yet mated. At this time point, half of the fish carrying IoA-00 lice and half of the fish carrying IoA-10 lice were removed from tanks to harvest lice required for the setup of batch crosses. The remaining fish in IoA-00 and IoA-10 tanks, as well as all fish carrying IoA-02 lice, were maintained further, allowing lice of the respective strains to mate and produce eggs. In the first batch cross the P0 generation consisted of IoA-00 males and IoA-10 females, whereas the second batch cross was set up with the inverse sex-strain orientation. Per cross, ten Atlantic salmon smolts were anaesthetised using 2-phenoxyethanol (100 mg/L; 99%; Sigma-Aldrich, Gillingham, UK) and each fish received two male and three female parasites. The two crosses, as well as the three tanks with strains IoA-00, IoA-02 and IoA-10, were maintained until dams produced egg strings, at which point they were removed, and egg strings incubated to produce copepodids. The resulting F1 larvae were used to inoculate tanks containing naïve Atlantic salmon. Infections were maintained until F1 parasites reached the adult male and preadult-II female stages. Subsequently, F1 parasites were subjected to traditional DTM bioassays to determine their susceptibility phenotype (see below).

### *Lepeophtheirus salmonis* bioassays

Traditional bioassays involved several DTM concentrations and had the purpose to characterise the DTM susceptibility of a parasite population or strain by estimating the compound’s median effective concentration (EC_50_). Single-dose bioassays used one diagnostic DTM concentration and served to characterise the susceptibility phenotype of individual lice. Bioassays with host-associated *L. salmonis,* i.e. adult stage males and preadult II stage or non-gravid adult stage females, had either a traditional or a single-dose design^30,33^, while all bioassays with copepodid larvae had a traditional design. In addition, exposure experiments to assess effects of DTM on whole body ATP levels were carried out. ATP experiments followed a design identical to that of single-dose bioassays, except that these trials were restricted to male parasites, used different exposure times to capture early physiological effects (see below), and included in addition to DTM one concentration of the acaricide fenpyroximate that is known to act by interference with the mitochondrial complex I^34^ and has been to shown deplete ATP levels in *L. salmonis*^21^. DTM and fenpyroximate (Pestanal analytical standard grade, Sigma-Aldrich, Gillingham, UK) were initially dissolved in acetone, with further dilutions carried out in a way to reach a final solvent concentration of 0.05% (v/v). All bioassay incubations took place in a temperature-controlled chamber set to 12 °C. Bioassays were considered invalid if the number of affected animals in solvent controls exceeded 10%. After completion of bioassays, test animals were stored in absolute ethanol at − 20 °C pending DNA extraction and genetic analyses.

In bioassays with host-associated *L. salmonis* stages, animals were introduced to 300 mL crystallising dishes containing 100 mL of filtered (55 µm) seawater equilibrated to 12 °C. After allocation of test animals to vessels, dishes were randomly assigned to treatments. Traditional bioassays included a geometrical series of at least six DTM concentrations in the range of 0.125 and 32 µg/L and a solvent control (Supplementary Table S2) in duplicates, with each dish receiving about five females and five males. In single-dose bioassays, lice were exposed to 2 µg/L DTM, which is the concentration recommended for treatments^30^. Each dish contained about ten females and ten males. Both types of bioassays further included solvent controls and involved exposure of lice for 30 min followed by recovery in clean seawater for 24 h prior to behavioural responses being examined and lice being rated as “live”, “weak”, “moribund”, or “dead”, with these criteria having been defined previously^29^. Animals rated as live or weak were considered unaffected, while those rated moribund or dead were considered affected by treatments. Lice were considered DTM resistant if remaining unaffected after exposure to 2 µg/L DTM and recovery, and susceptible to DTM if found affected.

*Lepeophtheirus salmonis* copepodid bioassays were carried out using batches of larvae generated by incubation of paired egg strings derived from the same dam (see above). After the number of copepodids per unit volume of seawater had been ascertained by counting, approximately ten copepodids were added to embryo dishes with glass lids by dispensing an appropriate volume of the batch, followed by topping up to a total volume of 2 mL with filtered seawater. Chemical exposures were initiated by adding 1 µL of a 2000 × final concentration solution of DTM to dishes containing 2 mL seawater and parasites. Copepodids were exposed to a geometric series of at least four concentrations of DTM in the range of 0.10 to 100 µg/L and one solvent control, in duplicates. Exposures in larval bioassays were for 24 h, followed by examination and rating of the animals. Copepodids were rated “live” when attracted by light and swimming normally, “weak” when swimming irregularly (animals swim in a straight line within 2 min after stimulation using light and a fine brush), “moribund” when incapable of swimming away after stimulation by light and a fine brush (animals may twitch appendages), and “dead” when showing no movements in extremities, gut, or other organs as apparent from examination under a microscope. Copepodids rated live or weak were considered unaffected, while moribund and dead parasites were considered affected.

In order to assess drug effects on whole body ATP levels, an exposure experiment was designed, with exposure levels and periods being based on similar experiments reported earlier^21^. Male lice were exposed to DTM for 30 min followed by recovery in clean seawater for 300 min. Exposure to fenpyroximate was for 300 min, without seawater recovery. After drug exposure, and where applicable seawater recovery, behavioural responses of animals was rated according to categories given above, and animals deemed “live”, “weak” or “moribund” were removed for ATP analyses.

### Effects of DTM on *L*. *salmonis* ATP levels

Directly after experimental exposure and rating of behavioural responses, individual lice were added to plastic tubes containing 1 mL of Tris–EDTA-saturated phenol (10 mM Tris HCl and 1 mM EDTA; Sigma-Aldrich, Gillingham, UK; Thistle Scientific Ltd, Glasgow, UK). Samples were incubated at room temperature for 10 min and subsequently stored at − 70 °C pending ATP analyses. Whole-body ATP levels were measured using a commercially available luciferin-luciferase bioluminescence assay kit (A-22066, Molecular Probes, Thermo Fisher Scientific, Bishop's Stortford, UK), with details having been reported elsewhere^21^.

### Extraction of genomic DNA

Genomic DNA (gDNA) was extracted from individual preadult-II or adult stage *L. salmonis* using a salt extraction method described in detail elsewhere^21^, producing high quality gDNA suitable for use in PCR reactions to amplify the mtDNA genome or genotyping assays. Alternatively, for samples only used in genotyping, DNA was extracted by a fast high throughput protocol^35^, with details having been reported elsewhere^25^. DNA from copepodids was extracted by the latter method and involved transfer of one specimen into a 0.2 mL tube containing 25 µL alkaline lysis buffer (25 mM NaOH, 0.2 mM EDTA, pH 12.0), followed by heating to 95 °C for 30 min and subsequently cooling to 4 °C for 5 min using a polymerase chain reaction (PCR) thermocycler. Then, 25 µL 40 mM Tris–HCL (pH 5.0) was added, and the sample vortexed briefly before being centrifuged at 4000×*g* for 1 min. DNA extracts were stored at − 20 °C pending use in genotyping analyses.

### Genotyping of mitochondrial single nucleotide polymorphism (SNP) alleles

PCR based genotyping assays employing universal fluorescence energy transfer (FRET) probes (KASP 4.0, LGC Genomics, Teddington, UK) were designed to detect 18 mtDNA SNPs that have shown association with DTM resistance in *L. salmonis* in a previous study^21^, namely four non-synonymous (G8134A, T5889C, T8600C, and G3338A) and 14 synonymous SNPs (A714G, G1174A, C1678T, C3056T, G4563A, G6325A, A9030G, A9426G, G10094A, TAG10722T, C11190,T G13466A, A14013G, C14751T) (Numbering according to NCBI Accession number LT630766.1). Each SNP assay involved one common primer and two allele specific primers (Supplementary Table S3), with further details being provided elsewhere^25^. Genotyping of preadult-II and adult *L. salmonis* collected at field sites and characterised by bioassays involved between 20 and 106 animals per site, while 20 to 24 parasites of previously characterised laboratory strains IoA-00, IoA-02 and NA01-O were genotyped. To characterise batches of larvae derived from one pair of egg strings, genotyping reactions were run for eight copepodids per batch.

### Mitochondrial haplotype network

A haplotype network was interfered from mitochondrial haplotypes of *L. salmonis*. The network was constructed using the medium-joining method^36^ implemented in the software PopArt v1.7^37^. Haplotypes were defined based on the combined occurrence of the 18 SNPs described above in preadult-II and adult *L. salmonis* collected at field sites for bioassay (see above).

### Mitochondrial genome (mtDNA) amplification and sequencing

The mitochondrial genome of two individuals of *L. salmonis* strain IoA-10 was amplified by PCR using gDNA as the template and sequenced. Amplification involved generating six overlapping amplicons using specific oligonucleotide primers designed with Primer3 v4.1.0 (Supplementary Table S4). PCR reactions were performed using 50 ng template DNA, 2.5 μL (10 pmol) each of forward and reverse primers, 25 μL Q5 High-Fidelity 2× Master Mix (New England BioLabs Ltd, Hitchin, UK), and 19 μL nuclease-free water. PCR conditions for each product are listed in Supplementary Table S5. All PCR products were purified (NucleoSpin Gel and PCR Clean-up Kit, Macherey–Nagel, Düren, Germany) and sequenced (Supplementary Table S6) by Eurofins Genomics (Ebersberg, Germany). Prior to sequencing, PCR product 6 (Supplementary Table S4) was subcloned using the pGEM-T Easy Vector system (Promega, WI, USA), followed by plasmid isolation using the NucleoSpin Plasmid EasyPure kit (Macherey–Nagel, Düren, Germany). MtDNA sequences obtained for the same individual were manually trimmed, aligned and assembled.

### Identification of DTM resistance-associated mtDNA SNP

MtDNA sequences from IoA-10 lice, as well as archived sequences from individuals of strains IoA-00 (N = 2), IoA-02 (N = 2), IoA-01 (N = 4), NA01-P (N = 2) and NA01-O (N = 3), wild hosts (N = 2), and F2 lice from IoA-00 and IoA-02 crosses (N = 12)^21^, were aligned to a *L. salmonis* mitochondrial genome assembly^21^ (NCBI Accession number: LT630766.1) using the R/Bioconductor package Rbowtie2 v 4.0.3^38^. Sequence variations were identified using the *HaplotypeCaller* function in GATK v3.5^39^. IoA-10 specific SNPs, as well as SNPs common to all DTM resistant individuals and lacking in all susceptible parasites were then identified.

### Sequence alignments

Amino acid sequences of ND1, ND5, COX1, and COX3 from *L. salmonis* and other crustaceans (GenBank accession numbers: *Caligus clemensis* HQ157566.1, *Caligus rogercresseyi* HQ157565.1*, Tigriopus caligornicus* DQ913891.2*, Paracyclopina nana* EU877959.1, *Calanus hyperboreus* NC_019627.1, *Eucalanus bungii* AB091772.1, *Squilla mantis* NC_006081.1, *Tetraclita japonica* NC_008974.1, *Homarus americanus* NC_015607.1, *Vargula hilgendorfii* NC_005306.1, *Daphnia pulex* NC_000844.1, *Artemia franciscana* NC_001620.1, *Triops cancriformis* NC_004465.1) were aligned using default parameters in the online software Clustal Omega v2.1^40^.

To compare DTM-associated SNPs reported in previous studies using different reference mtDNA genomes^21,22^, nucleotide sequences of the respective assemblies were aligned using Needle^21,41^. An earlier study reported nucleotide sequences for four mtDNA genes in *L salmonis salmonis* collected in Norway, Scotland, Canada and Russia in 2000 and 2002^28^. Sequences from the study (NCBI accession numbers: Cyt b, AY602223-AY602402; A6, AY602407-AY602586; COX1, AY602587-AY602766 and 16S rRNA, AY602770-AY602949) were aligned to a mtDNA assembly^21^ to derive SNP genotypes at mtDNA loci studied in this report (see above).

### Data analyses and statistical tests

All statistical tests were carried out using the software R (v4.0.2), with the significance level set at P < 0.05. Dose–response relationships in *L. salmonis* bioassays were assessed by probit analysis using the packages *drc* (v3.0-1) and *MASS* (v7.3-55), assuming a log-normal distribution of drug susceptibility. Based on the fitted models, EC_50_ and 95% confidence limits were derived and effects of sex and origin/strain on drug susceptibility assessed. Fisher's exact test of independence was used to examine whether DTM susceptibility phenotypes observed in single dose bioassays differed between mitochondrial haplotypes, employing the R package *rcompanion* (v2.4-1). Analyses of whole-body ATP data were conducted using the R packages *car* (v3.0-11) and *PMCMR* (v4.3). Normality and homogeneity of variances of data were assessed by Shapiro–Wilk’s and Levene’s tests, respectively. As the data violated these assumptions, the Kruskal–Wallis test was used to assess the effect of drug treatments on ATP levels, followed by *post-hoc* comparisons of treatments to the appropriate control group with Dunn’s test. The experiment-wise type I error was controlled by sequential Bonferroni correction^42^.

## Results

### Genotyping DTM resistant and susceptible *L. salmonis* at four SNP loci

Based on the combinations of genotypes at four non-synonymous mtDNA SNP loci expressed in *L. salmonis* samples tested, four haplotypes were defined (Table 1). Lice of strain IoA-00 (DTM-susceptible) possessed haplotype 1, defined by the wild type alleles T8600, G8134, T5889, and G3338, while lice of strain IoA-02 (DTM resistant) showed haplotype 2, comprising mutant alleles 3338A, 5889C, 8134A, and 8600C (Table 2). The majority (89.4%, N = 195) of *L. salmonis* collected from field sites (N = 218) were rated DTM resistant by bioassay. Across all lice obtained from field sites, 6.4% (N = 14) expressed haplotype 1, 72% (N = 157) haplotype 2, 14.2% (N = 31) haplotype 3 and 7.3% (N = 16) haplotype 4. The number of animals classified as DTM resistant did not differ between haplotypes 2 (94%), 3 (87%) and 4 (100%; P > 0.05), but was significantly greater for each of these haplotypes than for haplotype 1 (43%; P < 0.05; Table 2 and Supplementary Table S7).

**Table 1:** Haplotype definitions.

Position^a^	Alleles	Haplotype 1	Haplotype 2	Haplotype 3	Haplotype 4
3338	G/A	G	A	G	G
5889	T/C	T	C	T	C
8134	G/A	G	A	G	A
8600	T/C	T	C	C	C

Haplotypes 1, 2, 3, and 4 were defined based on the combined occurrence of the four non-synonymous single nucleotide polymorphisms (SNPs) that have been linked to resistance in a previous study^21^. G3338A, T5889C, G8134A, and T8600C are located in mitochondrial genes coding for NADH dehydrogenase subunit (ND1), NADH dehydrogenase subunit (ND5), cytochrome c oxidase subunit (COX1), and cytochrome c oxidase subunit 3 (COX3).

^a^Numbering according to a *L. salmonis* mitochondrial reference genome established for a Scottish isolate (NCBI Accession number LT630766.1).

**Table 2:** Association of mitochondrial haplotypes with deltamethrin resistance.

Origin of *L. salmonis*	Deltamethrin resistance^a^	N	Haplotype 1	Haplotype 2	Haplotype 3	Haplotype 4
IoA-00	Susceptible	22	22	–	–	–
IoA-02	Resistant	24	–	24	–	–
Farm site 1	Susceptible	3	1	2	–	–
(Argyll, 2018)	Resistant	103	3	76	16	8
Farm site 2	Susceptible	3	1	2	–	–
(Argyll, 2018)	Resistant	51	2	39	4	6
Farm site 3	Susceptible	7	–	4	3	–
(Argyll, 2019)	Resistant	21^b^	–	16	3	2
Farm site 4	Susceptible	10	6	3	1	–
(Sutherland, 2019)	Resistant	20	1	15	4	–
Total (farm sites)		218	N = 14	N = 157	N = 31	N = 16
	Susceptible	23	57.1%	7%	12.9%	0%
	Resistant	195	42.9%	93%	87.1%	100%

*L. salmonis* of laboratory strains IoA-00 and IoA-02 and parasites obtained from Scottish aquaculture production sites were subjected to deltamethrin bioassays to establish susceptibility status, followed by genotyping at four SNP loci to establish haplotypes (see Table 1 for details).

^a^Susceptibility to deltamethrin was determined in single-dose bioassays, involving exposure (30 min) to 2 µg/L deltamethrin, followed by recovery in seawater (24 h) and subsequent rating as susceptible (affected) or resistant (unaffected).

^b^21 out of a total of 37 deltamethrin resistant individuals observed were successfully genotyped.

### Relationship of mtDNA haplotypes to DTM resistance of copepodids

The relationship between mtDNA haplotypes and DTM resistance was further investigated in 24 h behavioural bioassays with copepodid larvae derived from gravid females collected at field sites or obtained from laboratory strain cultures. The estimated DTM EC_50_ of copepodids was 0.14 µg/L for haplotype 1 (strain IoA-00), 4.81 µg/L (strain IoA-02) or 3.98 µg/L (field derived larvae) for haplotype 2, and 6.90 µg/L for haplotype 3, field derived larvae (Table 3, Supplementary Data S1).

**Table 3:** Deltamethrin susceptibility and mitochondrial haplotype of *L.* *salmonis* copepodid larvae.

Origin of *L.* *salmonis*	Num bioassay	Haplotype^a^	EC_50_ (95% CI)^b^ [µg/L]
IoA-00	6	1	0.14 (0.13–0.16)
IoA-02	3	2	4.81 (3.99–5.63)
Farm site 5, 6	2	2	3.98 (2.93–5.04)
Farm sites 5, 7	2	3	6.90 (4.15–9.65)

Each bioassay was performed with the copepodid descendants of one female salmon louse. *L.* *salmonis* originated from the laboratory strains IoA-00 and IoA-02, or Scottish aquaculture production sites. Bioassays involved exposure (24 h) to deltamethrin and rating of copepodids as normal or affected. Haplotypes were established based on the genotyping of 10 individuals per bioassay.

^a^ For definition of haplotypes, see Table 1.

^b^Raw data used to derive EC_50_ values are provided in Supplementary Data S1.

### Genotyping DTM resistant and susceptible *L. salmonis* at 18 SNP loci

**Figure 1:**
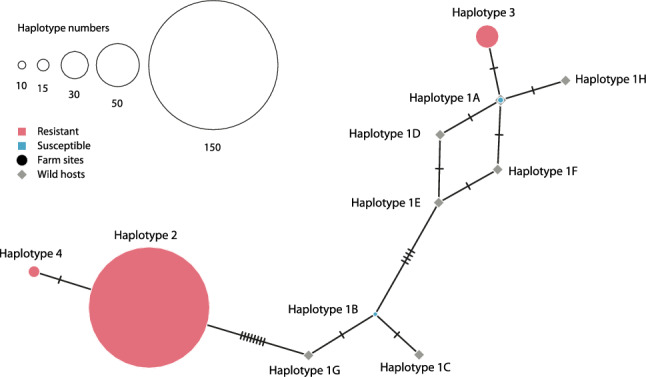
Median-joining haplotype network interfered from mitochondrial haplotypes of *L. salmonis*. Haplotypes were defined based on the combined occurrence of 18 mitochondrial SNPs, which have been associated with deltamethrin resistance in *L. salmonis* in a previous study^21^ (Supplementary Tables S8 and S9). Haplotypes that were identified in salmon lice from farm sites are represented by circles, while haplotypes that were identified in salmon lice from wild hosts are represented by grey rhombi. The size of each circle is proportional to the frequency of each haplotype in salmon lice from farm sites. Deltamethrin resistance associated haplotypes are represented by red circles and susceptibility-associated haplotypes are shown in blue.

A subset of the above sample, as well as selected archived historical samples, were characterised using a wider panel of SNPs that further included 14 non-synonymous mtDNA mutations. This allowed the above haplotype definition to be refined. Haplotypes 2, 3 and 4 defined above remained after taking into account the wider range of SNPs, while the originally defined haplotype 1 (Table 1) was subdivided into 8 haplotypes named 1A to 1H (Supplementary Table S8). Results of the expanded genotyping are summarised in Supplementary Table S9. For IoA-00 and IoA-02 strain lice, as well as for lice from sites 1 to 3, results with the wider set of SNP assays are similar to those reported above (Table 2) based on only non-synonymous SNPs. Lice collected from wild hosts in 2010 expressed, in addition to putative susceptible haplotypes 1A and 1C to 1H, the resistance-associated haplotype 4. Strain NA01-O lice contained resistance-associated haplotypes 2, 3 and 4. The phylogenetic analysis of the 11 haplotypes obtained within this study revealed that resistance-associated haplotype 3 was closely related to susceptible haplotypes (1A, 1D-F, and 1H), but phylogenetically distant to resistance-associated haplotypes 2 and 4 (Fig. 1). Compared to susceptibility-associated haplotype 1A expressed in strain IoA-00, the resistance-associated haplotype 3 differed in genotype at only one of 18 tested SNP loci, T8600C in COX1 (Supplementary Table S8).

### *Lepeophtheirus salmonis* mtDNA haplotypes observed in earlier studies

Re-analysis of mtDNA sequence data reported in a previous study of Atlantic *L. salmonis* populations sampled in 2000 and 2002^28^ allowed deriving genotypes for 8 of the SNPs studied in this report and assign samples to mtDNA haplotypes (Supplementary Data S2). All lice of the historical data set (N = 180) showed the wild-type thymidine nucleotide at position 8600. The most frequent mtDNA haplotypes were 1A (N = 140; 77.8%) and 1B (N = 21; 11.7%), while DTM resistance-associated haplotypes 2 to 4 of this report were lacking. Less frequent haplotypes of historical samples included 1D (N = 5; 2.8%), (N = 4; 2.2%) and 1E/1F (together N = 2; 1.1%), as well as new haplotypes 1I-L (together N = 8; 4.4%) (Supplementary Data S2).

A previous study has derived mtDNA SNPs putatively associated with DTM resistance after characterising lice generated in a crossing experiment involving DTM resistant and susceptible *L. salmonis* isolates collected in Norway^22^, with mutations reported including all SNPs studied in this report except for those at positions 10722 and 13466. Re-analysis of data from the study allow to assign DTM resistant lice of the study to haplotype 2 and DTM susceptible lice to haplotypes 1A or 1D defined in the current report (Supplementary Data S3).

### Establishment and characterisation of a *L. salmonis* strain expressing haplotype 3

Among gravid *L. salmonis* females (n = 30) obtained from a salmon production site in November 2019 and subjected to genotyping, haplotypes 1 (N = 1), 2 (N = 22), 3 (N = 2) and 4 (N = 5) were represented. The offspring of one of the females of haplotype 3 was used to establish *L. salmonis* laboratory strain IoA-10. The mitochondrial genome of IoA-10 was sequenced (EBI ENA project reference: PRJEB47839), revealing 36 strain-specific sequence variations that were absent in previously studied strains^21^ IoA-00, IoA-01, IoA-02, IoA-03, NA01-O, and NA01-P (Supplementary Table S10). Comparison of the mtDNA genome of IoA-10 strain lice to that of DTM resistant and susceptible *L. salmonis* strains (IoA-00, IoA-01) revealed a limited number of mtDNA mutations that were present in all DTM resistant lice while lacking in all analysed susceptible isolates. These mutations comprised the non-synonymous SNP T8600C, corresponding to Leu107Ser in COX1, and eight polymorphisms in regions that are non-protein coding (Table 4).

**Table 4:** Mitochondrial sequence variations specific for deltamethrin resistant *L. salmonis* isolates.

Position^a^	Type	Location	Description	Reference	Alternative
812	Polymorphism	D-loop		T	C
875	Polymorphism	D-loop		T	C
940	Deletion	D-loop		AG	A
960	Polymorphism	D-loop		T	C
963	Polymorphism	D-loop		G	A
972	Polymorphism	D-loop		A	G
8600	Non-synonymous mutation	COX1	TTG/Leu → TCG/Ser	T	C
10178	Polymorphism	l-rRNA		A	G
15377	Polymorphism	D-loop		G	A

The mitochondrial genome (mtDNA) was compared between deltamethrin resistant (IoA-02, IoA-03, IoA-10, NA01-O, NA01-P) and susceptible (IoA-00, IoA-01) *L. salmonis* strains to establish mutations associated with resistance. The mtDNA sequence of strain IoA-10 was established by PCR followed by Sanger sequencing within the present study, while that of the other strains has been reported previously^21^.

^a^Numbering according to a *L. salmonis* mitochondrial reference genome established for a Scottish isolate (NCBI Accession number LT630766.1).

**Figure 2:**
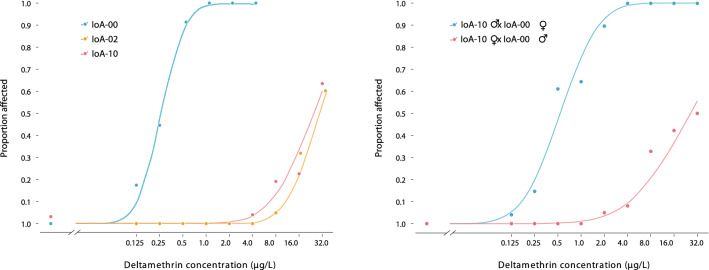
Traditional deltamethrin bioassay with *L. salmonis* first filial (F1) progenies derived from parental (P0) crosses of different gender-strain orientations. Median effective concentrations EC_50_ [µg/L] and 95% confidence limits: IoA-10 = 24.73 (15.06–34.39), IoA-00 = 0.25 (0.20–0.30), IoA-02 = 25.95 (17.98–33.92), F1: IoA-10 dam x IoA-00 sire = 26.10 (11.46–40.75), F1: IoA-10 sire x IoA-00 dam = 0.55 (0.41–0.70). Bioassays involved exposure (30 min) to deltamethrin, followed by recovery in seawater (24 h) and rating of lice as normal or affected. Dose response relationships were established for F1 females and males combined as sex differences were not significant (P > 0.05). Raw data are provided in Supplementary Data S4.

Reciprocal crosses between strains IoA-00 and IoA-10 produced two sets of F1 lice (Supplementary Table S11), which were assessed in DTM bioassays along with synchronised IoA-02 and parental strain parasites. Strains IoA-02 and IoA-10, as well as F1 offspring derived from IoA-10 dams and IoA-00 sires, were highly DTM resistant (EC_50_ values > 24.0 µg/L; Fig. 2, Supplementary Data S4). In contrast, F1 animals derived from IoA-00 dams and IoA-10 sires (EC_50_ 0.55 µg/L; Fig. 2) were only marginally but significantly (P < 0.0001) less susceptible to DTM than IoA-00 lice (EC_50_ 0.25 µg/L; Fig. 2).

**Figure 3:**
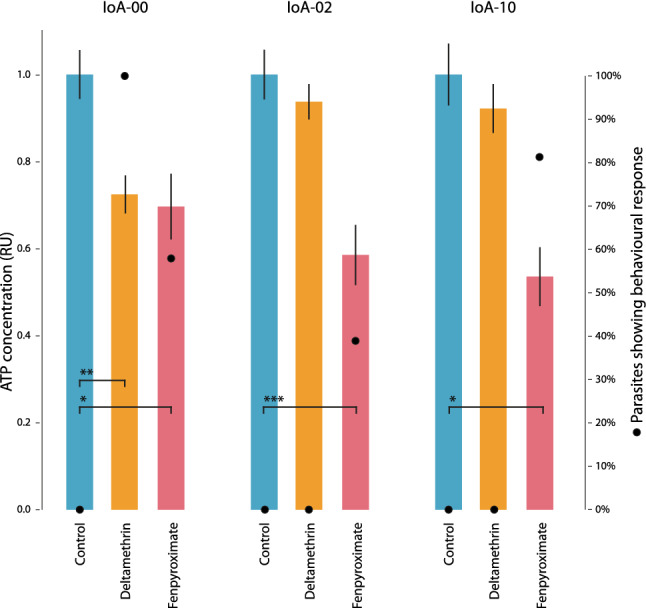
Effect of deltamethrin and fenpyroximate on ATP levels in *L. salmonis*. Male adult salmon lice of the drug susceptible strain IoA-00, the multi-resistant strain IoA-02, and strain IoA-10 were exposed to deltamethrin (2 µg/L), fenpyroximate (100 µg/L), or a solvent acetone control (0.05% v/v, control) for 300 min before behavioural effects were recorded (secondary y-axis; orange data points) and alive animals were sampled for whole-body ATP analysis (N = 15 per group). ATP concentrations in drug treated lice are expressed relative to those of the control group (relative units, RU ± SE). Stars indicate significant differences to the control group (Kruskal–Wallis test followed by post hoc comparisons using Dunn’s test; * P < 0.05, ** P < 0.01, *** P < 0.001).

Exposure of lice to fenpyroximate (100 µg/L), an acaricide known to block oxidative phosphorylation, caused toxicity, and decreased whole-body ATP levels in all strains assessed (Fig. 3, Supplementary Tables S5 and S12). Exposure to 2 µg/L DTM caused toxic effects accompanied by significantly decrease of ATP levels in IoA-00 lice (P = 0.008) but failed at causing toxicity or significant effects on the ATP levels in IoA-02 and IoA-10 lice.

### Protein sequence comparisons of ND1, ND5, COX1, and COX3 among crustacean species

Amino acid sequences of ND1, ND5, COX1, and COX3 from *L. salmonis* and different crustacean species for which mtDNA sequences were available were aligned to reveal conserved residues within these mitochondrial polypeptides. Among four non-synonymous mtDNA SNP loci that have previously been reported from a DTM resistant strain^21^, G3338A (COX3 Gly33Glu), T5889C (ND5 Leu411Ser) and G8134A (ND1 Gly251Ser) cause residue changes at non-conserved positions, while T8600C (COX1 Leu107Ser) alters the amino acid sequence at a position that is conserved among all species assessed (Supplementary Fig. S1).

## Discussion

Previous studies have shown that DTM resistance in *L. salmonis* is transmitted predominantly by maternal inheritance and associated with mtDNA SNPs^20,21^. While an earlier study suggested that highly DTM resistant *L. salmonis* share virtually identical mtDNA sequences^21^, the present study identified three mtDNA haplotypes associated with resistance, one of which coincides with the previously reported resistance associated mtDNA sequence. *L. salmonis* strain IoA-10, which possesses one of the novel DTM resistance associated haplotypes, showed a high level of DTM resistance comparable to that of the previously characterised strain IoA-02 and, similar to IoA-02, passed on DTM resistance to the next generation through maternal inheritance. The phylogenetic analysis of mtDNA haplotypes provided evidence for multiple origins of mtDNA-associated DTM resistance in *L. salmonis*. Comparison of mtDNA sequences between DTM resistant and susceptible salmon louse strains suggested the association of DTM resistance with SNP T8600C, corresponding to Leu107Ser in COX1.

*Lepeophtheirus salmonis* obtained at aquaculture sites were subjected to bioassays to determine their DTM susceptibility status. Parasites were then genotyped at previously described mtDNA SNP loci^21^ to identify mitochondrial haplotypes and assess haplotype association with DTM resistance. 93%, 87%, and 100% of lice possessing haplotypes 2, 3, and 4, respectively, were classified as DTM resistant, emphasising the association of mtDNA mutations with the resistance phenotype. The remaining 7–13% may have died due to interacting environmental factors or handling of parasites during sampling, transportation, and set-up of bioassays rather than drug toxicity^13,43^. As bioassays were performed on lice directly obtained from farmed salmon, environmental conditions prior sampling could not be controlled. Lice may have also been exposed to stressful conditions during sampling and due to abrupt changes of environmental conditions during transportation. The DTM resistance phenotype was less distinct for lice containing haplotype 1. While most lice with this haplotype were classified as DTM susceptible, 43% were rated resistant. The large number of resistant haplotype 1 individuals suggests the contribution of genetic factors other than mitochondrial mutations to the DTM resistance phenotype, which is in line with findings by Carmona-Antoñanzas et al.^21^ who attributed the presence of about 20% resistant F2 parasites in a family descending from a DTM susceptible dam and a resistant sire to nuclear genetic determinants of resistance.

To further assess the relationship between mtDNA haplotypes and DTM resistance, bioassay experiments were carried out with batches of copepodid larvae derived from individual females. Compared to bioassays with field collected preadult-II and adult lice, advantages of F1 bioassays with copepodid larvae include the fact that sibling clutches share the same mitochondrial haplotype^44^ and were hatched and reared under standardized laboratory conditions less likely to bias the outcome of the experiment. While insufficient gravid females of appropriate genotype precluded analysis of haplotype 4 in copepodid bioassays, testing of larvae of the remaining haplotypes resolved significant differences in DTM susceptibility between *L. salmonis* of haplotypes 2 and 3 as compared to parasites of haplotype 1, confirming results from conventional bioassays and underpinning the association of mtDNA mutations with DTM resistance. Moreover, the study showed that for DTM resistant lice, hyposensitivity to the drug is already apparent at the larval stage, which would be expected for resistance conferred by mtDNA mutations^21^.

Genotyping of field isolates collected in 2018 and 2019 revealed three DTM resistance-associated mtDNA haplotypes, with haplotypes 2, 3, and 4 being found in 75%, 14% and 8% of all resistant lice, respectively. While an earlier study from our laboratory^21^ found that all Scottish DTM resistant isolates shared mtDNA sequences consistent with haplotype 2, re-analysis of a larger number of archived samples from these isolates during this study provides evidence for haplotypes 2, 3 and 4 being present in DTM resistant strain NA01-O, which was established in 2013, while haplotype 4 was expressed by some specimens among a sample of lice removed from wild salmon in 2010. In contrast, re-analysis of sequence variation data for four mitochondrial genes in *L. salmonis* populations from Norway, Scotland, Canada and Russia sampled in 2000–2002^28^ failed to detect in any of the samples the DTM resistance-associated mutation Leu107Ser in COX1, which is shared among the three DTM resistance associated mtDNA haplotypes identified in this report. In accordance with these findings, another study, which scanned *L. salmonis* populations from sites across the North Atlantic for one DTM resistance associated mtDNA SNP^45^, found that the resistance-associated genotype first appeared in 2009 in populations from Shetland, Norway and Ireland, but was absent in samples from 2002 and before. Phylogenetic analyses of mtDNA haplotypes identified in this study showed that the three DTM resistance-associated haplotypes fall into two clusters, with haplotype 3 being phylogenetically distant to haplotypes 2 and 4, which shared 17 out of 18 tested SNP loci. This suggests that mtDNA-associated DTM resistance in *L. salmonis* evolved at least twice independently followed by spread of resistant lice as a consequence of the extensive use of pyrethroids. However, more research is required to resolve at greater detail the roles of different DTM resistance associated mtDNA haplotypes in the emergence and spread of *L. salmonis* pyrethroid resistance across the North Atlantic.

Resistance-associated haplotypes 2 and 3 differed maximally in sequence, with only one out of 18 tested SNP loci being shared. However, despite haplotypes 2 and 3 being very different in sequence, the resistance phenotype conferred is very similar. Both IoA-10 (haplotype 3) and IoA-02 (haplotype 2) lice were highly DTM resistant (EC_50_ values > 24.0 µg/L; P = 0.845) and reciprocal crosses of strains IoA-10 (present study) and IoA-02^21^ with the drug-susceptible IoA-00 lice revealed that both strains transmit their resistance to the next generation through maternal inheritance. Moreover, DTM exposure caused behavioural toxicity and whole-body ATP depletion in DTM susceptible IoA-00 parasites, but not resistant IoA-10 and IoA-02 lice. These findings are in line with an earlier experiment, which compared the effect of DTM exposure on behavioural toxicity and ATP levels between IoA-00 and IoA-02 lice^21^. Depletion of ATP levels in DTM susceptible lice may be related to the toxic effect of DTM on the mitochondria, and mtDNA mutations in haplotypes 2 and 3 may have a protective effect.

As the inheritance of mtDNA is linear and lacks recombination through meiosis, relevant SNPs for DTM resistance are transmitted together with irrelevant hitchhiking SNPs^44^. Thus, SNPs that are truly linked to DTM resistance are expected to be present in all resistance-associated haplotypes but lacking in all susceptibility-associated haplotypes. The non-synonymous mtDNA SNP T8600C, corresponding to Leu107Ser in COX1, was the only mutation shared by the resistance-associated haplotypes 2, 3, and 4 and lacking in all susceptibility-associated haplotypes. When comparing mtDNA sequences of DTM resistant and susceptible lice, T8600C was also the only non-synonymous mutation differentiating between resistant and susceptible individuals. Sequencing analyses further revealed eight additional SNPs in non-coding regions (NCR) of the mtDNA that were common to all resistant lice and lacking in all susceptible lice. NCR sequences are the most variable mtDNA sequences, which may explain the high number of SNPs found within this region in the present study^46^. Seven of these SNPs were found in the mitochondrial control region, also known as displacement loop (D-loop). Its function is not yet fully understood but seems to be critical in regulating replication and transcription of mtDNA^47^. However, D-loop mutations are not known to confer drug resistance. Another SNP, A10178G, was found within a mitochondrial ribosomal RNA (rRNA) gene and has also been described by Bakke et al.^22^. Mitochondrial rRNAs are assembled with ribosomal proteins encoded by nuclear genes to form mitochondrial ribosomes, which are responsible for translating mitochondrial proteins^48^. Thus, mutation within the mitochondrial rRNA may lead to ribosome dysfunction and may result in respiratory chain defects^49^. However, to our knowledge, there are no reports of mitochondrial rRNA mutations associated with drug resistance.

Findings of the present study raise questions about the mechanism of DTM resistance and by inference the mechanism of DTM toxicity in *L. salmonis.* While it is generally accepted that pyrethroids target Na_v_ in terrestrial arthopods^16^, several studies with terrestrial arthropods and mammals provide evidence for pyrethroid effects on mitochondrial functions. Due to their lipophilic nature, pyrethroids can pass and interact with biological membranes, making mitochondrial membranes and membrane proteins candidate targets for toxic action^50^. For example, pyrethroids have been shown to affect mitochondrial membrane structures and dynamics, which can impair oxidative phosphorylation^51,52^. Mitochondrial oxidative phosphorylation can also be impaired by intracellular Ca^2+^ accumulation^53,54^, which can result from interactions of pyrethroids with Na_v_ and consequent Ca^2+^ influx^55^ and direct effects of pyrethroids on voltage-gated Ca^2+^ channels^56^. Pyrethroid induced disruption of mitochondrial membrane integrity and inhibition of respiratory complexes, as well as intracellular Ca^2+^ accumulation can cause the generation of reactive oxygen species (ROS) in mitochondria^57–59^. ROS can trigger a cascade of reactions that induces lipid peroxidation and damage of macromolecules^60–62^.

Pyrethroids have been shown to induce intrinsic mitochondrial apoptosis^63,64^, which involves mitochondrial outer membrane permeabilization, release of cytochrome C into the cytosol, activation of caspases, and ultimately DNA fragmentation^65^. In particular, this pathway can be triggered by pyrethroid induced oxidative stress and Ca^2+^ accumulation, as well as low levels of ATP that lead to disruption of the mitochondrial transmembrane potential^66–69^. Interestingly, DTM exposure increased apoptosis in mitochondria-rich skeletal muscle, subcuticular tissue, and central ganglion cells in salmon lice of a drug susceptible strain, but not or to a lesser degree in a DTM resistant strain^22^.

Taken together, in salmon lice, DTM may induce toxicity through disruption of mitochondrial membranes, direct inhibition of mitochondrial respiratory complex(es), intracellular Ca^2+^ accumulation, or by causing oxidative stress. An obvious explanation for DTM toxicity in salmon lice would be that DTM or its metabolites are binding to a mitochondrial respiratory complex and lead to disruption of the mitochondrial ATP production, which has been observed in the present study. In addition, inhibition of respiratory complexes can lead to the formation of ROS^57^. Both, low levels of ATP and oxidative stress can in turn induce intrinsic mitochondrial apoptosis^66,67,69^, which has been described by Bakke et al.^22^. Resistance may be conferred by mitochondrial SNP(s) that changes the amino-acid sequences of the complex and impair binding of DTM. For reasons explained above, T8600C leading to Leu107Ser in COX1 is the most probable mutation for conferring DTM resistance in salmon lice. Alternatively, DTM might impair the mitochondrial ATP production in susceptible lice by causing disruptions of the mitochondrial membrane or by secondary effects arising from DTM toxicity. In these scenarios, mtDNA mutation(s) may have functional effects on the efficiency of electron transfer or proton translocation, counteracting ATP deficits.

While the association of DTM resistance with mtDNA mutations is well documented in *L. salmonis*^20–22^*,* implying mitochondrial targets for DTM toxicity, evidence for roles of mitochondrial genes in pyrethroid resistance is lacking in other arthropods. If point mutations of mtDNA are sufficient to confer DTM resistance in *L. salmonis,* a possibility suggested by this and previous studies, this would further imply that Na_v_ is of secondary importance in the mode of action of DTM in *L. salmonis.* Interestingly, DTM toxicity in *L. salmonis* showed a delayed onset in earlier studies. In bioassays, no effects of DTM on lice of a susceptible strain were apparent directly after 30 min of exposure to 0.5 µg/L DTM and three hours of recovery in clean seawater were required for effects to fully develop^21^, while in experimental treatments of fish carrying susceptible strain lice, the median survival time of parasites after a 30 min bath treatment with 2.0 µg/L DTM was 16.8 hours^70^. While the delayed onset of DTM toxic effects observed in these studies is unexpected for a drug assumed to cause disruption of neurotransmission, more research is required to characterise the molecular mode of action of DTM in *L. salmonis*.

## Conclusion

DTM resistance in *L. salmonis* is associated with multiple mtDNA haplotypes which have multiple origins. Non-synonymous mtDNA mutation T8600C, corresponding to Leu107Ser in COX1, was common to all DTM resistant mtDNA haplotypes but lacking in haplotypes not associated with resistance. Parasites possessing a mtDNA haplotype in which T8600C was the only non-synonymous mutation are highly DTM resistant and pass on their resistance to the next generation through maternal inheritance. The results suggest the association of DTM resistance with SNP T8600C (Leu107Ser in COX1).

## Supplementary Information

Supplementary Information 1.

Supplementary Information 2.

Supplementary Information 3.

Supplementary Information 4.

Supplementary Information 5.

Supplementary Information 6.

## Data Availability

The sequencing data were submitted to the EBI ENA database under the Project number PRJEB47839.
