# Supplementary material for: Key role of mitochondrial mutation Leu107Ser (COX1) in deltamethrin resistance in salmon lice (Lepeophtheirus salmonis)
Source: Sci Rep. 2022 Jun 20;12:10356. doi: 10.1038/s41598-022-14023-1 (PMC9209418; doi:10.1038/s41598-022-14023-1)
Supplement: Supplementary file 1 — Supplementary Information 1. [file 41598_2022_14023_MOESM1_ESM.pdf]

Key role of mitochondrial mutation Leu107Ser (COX1) in deltamethrin resistance in salmon lice (*Lepeophtheirus salmonis*)

Claudia Tschesche[1], Michaël Bekaert[1], David I. Bassett[1], Sally Boy[1], James E. Bron[1], and Armin Sturm[1]

[1]Institute of Aquaculture, Faculty of Natural Sciences, University of Stirling, Stirling, FK9 4LA, United Kingdom

ABSTRACT

The pyrethroid deltamethrin (DTM) is used to treat Atlantic salmon (*Salmo salar*) against salmon louse (*Lepeophtheirus salmonis*) infestations. However, DTM resistance has evolved in *L. salmonis* and is currently common in the North Atlantic. This study aimed to re-assess the association between DTM resistance and mitochondrial (mtDNA) mutations demonstrated in previous reports. Among 218 *L. salmonis* collected in Scotland in 2018-2019, 89.4% showed DTM resistance in bioassays, while 93.6% expressed at least one of four mtDNA single nucleotide polymorphisms (SNPs) previously shown to be resistance associated. Genotyping at a further 14 SNP loci allowed to define three resistance-associated mtDNA haplotypes, named 2, 3 and 4, occurring in 72.0%, 14.2% and 7.3% of samples, respectively. *L. salmonis* strains IoA-02 (haplotype 2) and IoA-10 (haplotype 3) both showed high levels (100-fold) of DTM resistance, which was inherited maternally in crossing experiments. MtDNA haplotypes 2 and 3 differed in genotype for 17 of 18 studied SNPs, but shared one mutation that causes an amino acid change (Leu107Ser) in the cytochrome c oxidase subunit 1 (COX1) and was present in all DTM resistant while lacking in all susceptible parasites. We conclude that Leu107Ser (COX1) is a main genetic determinant of DTM resistance in *L. salmonis*.

Supplementary Tables

Supplementary Table S1. *Lepeophtheirus salmonis* strains studied. All locations are in Scotland, UK. Concentrations are reported in µg/L.

Stain	Origin	Year isolated	Deltamethrin EC_{50} (95% conf.) [µg/L]
IoA-00	Firth of Clyde	2003	0.25 (0.20-0.30)∗
IoA-01	Sutherland	2008	0.36 (0.26-0.46)[1]
IoA-02	Shetland Islands	2011	25.95 (18.0-33.9)∗
IoA-03	Sutherland	2012	>2.0[1]
IoA-10	Argyll and Bute	2019	24.73 (15.06-34.39)∗
NA01-O	Argyll and Bute	2012	24.8 (12.2-85.7)[1]
NA01-P	Argyll and Bute	2012	80.5 (32.5-61.4)[1]

∗ Data produced in this study.

Supplementary Table S2. Deltamethrin concentrations (µg/L) used in bioassays *L. salmonis* with first filial (F1) progenies derived from parental (P0) crosses of different sex-strain orientations.

P0 generation	0.0	0.125	0.25	0.5	1.0	2.0	4.0	8.0	16.0	32.0
IoA-10 dam × IoA-10 sire	•	•	•	•	•	•	•	•	•	•
IoA-00 dam × IoA-00 sire	•	•	•	•	•	•	•			
IoA-02 dam × IoA-02 sire	•				•	•	•	•	•	•
IoA-00 dam × IoA-10 sire	•	•	•	•	•	•	•	•	•	•
IoA-10 dam × IoA-00 sire	•	•	•	•	•	•	•	•	•	•

Supplementary Table S3. *L. salmonis* primer sequences used for allele-specific PCR assays.

Name	Primer for allele 1 (FAM)	Primer for allele 2(HEX)	Common primer	Allele 1	Allele 2
3338	AATAGGCCTAAAGCCACAAACC	CCTAATAGGCCTAAAGCCACAAACT	GTCTCCTTGGCCTCTATTAAGGTCTT	G	A
5889	AAAGTGGGAAGTGTATCAAGGCTTTT	AAGTGGGAAGTGTATCAAGGCTTTC	CTGGTAAAATTTAAAAGACCCTCCTCAATT	T	C
8134	TTTGTTTTATGTAATTAGAAGGTTAGGAG	ACTTTTGTTTTATGTAATTAGAAGGTTAGGAA	TAACCCGCCTTCACACTCACAAGAA	G	A
8600	ACTAATGCCCTTATAAGTAATAAACTCA	ACTAATGCCCTTATAAGTAATAAACTCG	CCCCGCTTAAACAATATAAGATTTTGGTTT	T	C
714	GAGCATGTCATGAAAAGTCTAGGAGA	AGCATGTCATGAAAAGTCTAGGAGG	CACACAACTGCTCAGAGGAAGGATT	A	G
1174	CTACTATAACTTTACTCAATTTATTCCTC	CTCTACTATAACTTTACTCAATTTATTCCTT	TTAAGGCAATCGAGATTGGAAGTAGGAAA	G	A
1678	ATAGACCCCATAAGAGGGGCC	ATAGACCCCATAAGAGGGGCT	TACATTTATGACTTCCCTTAGCTCACGTA	C	T
3056	GTGTTATACTGTTGTAGGGTTATACTC	GAGTGTTATACTGTTGTAGGGTTATACTT	GTTCGTCTTCCGATTAATTTCAGCTGTAA	C	T
4563	CACACCTTAAGTATCTTTCTACCCTC	ACACACCTTAAGTATCTTTCTACCCTT	TTTCTAAGTGTAGGAGGAGTGGGGA	G	A
6325	GAGGAACAATTACCCCCTGAGC	GAGGAACAATTACCCCCTGAGT	GGTAACTTAAGGTATTCTGAACTAATCTTA	G	A
9030	TTATATTCTAATTCTTCCAGGGTTTGGA	ATATTCTAATTCTTCCAGGGTTTGGG	GCCTCATCTTTACAAGTTTCTTGGGTAAT	A	G
9426	CCACTTTCACTATGTTCTTAGAATAGGA	CACTTTCACTATGTTCTTAGAATAGGG	AGTGAACCCCGCTATTAGGGCAAAA	A	G
10094	CCTATGTGTAGCAAAATAATTGGTTCAG	ACCTATGTGTAGCAAAATAATTGGTTCAA	GGCTGGGATAGCCCACTTAATATAATAAA	G	A
10722	CCGATTAAAAATATCTATCCTCTCTCTC	CCCGATTAAAAATATCTATCCTCTCTCTA	CGTGTTCATCCTTGTCTTATAGCTTAGTT	TAG	T
11190	CCTCATGAAATGTTAATTTTTGAGATTTAAATG	CCTCATGAAATGTTAATTTTTGAGATTTAAATA	GTGCCAGCATTCGCGGTTATACTTT	C	T
13466	TAATCCGACCTCTAACCCTAAGG	GTTAATCCGACCTCTAACCCTAAGA	GCCTGCAATTATATTAGCTATAAGCCGAA	G	A
14013	TCTTTTATCCCCCAGCAAAAATGGA	CTTTTATCCCCCAGCAAAAATGGG	GTAGTGGCTTTAGCTTTGTCTGTAAGAAT	A	G
14751	CTACGGATAAAAACGCTGTAGAAATC	GTCTACGGATAAAAACGCTGTAGAAATT	CCGGAATTTTTTTGGCTATGCATTATTCAA	C	T

Supplementary Table S4. PCR primers used to amplify *L. salmonis* mtDNA sequences in six overlapping products (including PCR conditions).

PCR product	Forward primer (5' to 3')	Reverse primer (5' to 3')	Annealing temperature	Extension time	PCR program	Amplicon size (nt)
1	GGGGAAAGGGGGCTATCTACTTG	TTGCTCCCGCTAACACTGGTAAA	69°C	3 min	A	5749
2	TGTCTCAGCCGGGAGCATATTTA	GCGTAATAACTGCCCGCATTACA	67°C	3 min	A	5373
3	CAAAGGTTTGCCCACCTAGTTCC	CCAAACTCCGCGGTTACTACAGA	50/52°C	3 min	B	1810
4	AAGCTTAGCTAATACCAGGT	ATGGTGGCTGTTACCAAAAT	56/58°C	4 min	B	3508
5	TTAGCTCACAATCCGTCTGA	TTAGGCCCAAACTTTTCTACT	60°C	52 s	A	1634
6	AGTAGAAAAGTTTGGGCCTAAA	ATATTTCTCGCCGCTGGTATATTTT	61°C	65 s	A	1890

Supplementary Table S5. PCR conditions for each product

PCR program	Step	Temperature	Time	Cycles
A	Initial denaturation	98°C	30 s	
A	Denaturation	98°C	10 s	
A	Annealing	See Table S3	20 s	33
A	Extension	72°C	See Table S3	
A	Final extension	72°C	2 min	
B	Initial denaturation	98°C	2 min	
B	Denaturation	98°C	20 s	
B	Annealing	See Table S3	30 s	10
B	Extension	72°C	See Table S3	
B	Denaturation	98°C	20 s	
B	Annealing	See Table S3	30 s	20
B	Extension	72°C	See Table S3	
B	Final extension	72°C	2 min	

Supplementary Table S6. Oligonucleotide primers to sequence above PCR products.

PCR product	Primer ID	Sequencing primer (5' to 3')
1	XL-PCRF1	GGGGAAAGGGGGCTATCTACTTG
	XL-PCR-R1ii	ATCTACTGAAGCCCCGGAGT
	mtDNA_Seq63F	GTTAGATGTTTCGGTTGAG
	mtDNA_Seq66F	TCTCAGTTTAGTTTCCAAGA
	mtDNA_Seq43R	CCGCTAGACCTGTTAGGAAT
	mtDNA_Seq44R	GCACAGCGTCTATTTTAACC
	mtDNA_Seq45R	TGTGAGGAATAGGGAGTTTG
	mtDNA_Seq3R	CTCCCGGAAGTAACTCAGAA
	mtDNA_Seq27R	CCTGCAGTAACCAAAGTTGA
	mtDNA_Seq26R	ATTGTACCACATCCCCTCTG
	mtDNA_Seq25R	CCCCCTTCGTCTTAAAGAAT
	CT_Seq1.1R	ACGGATCAGGGGAATATTG
	CT_Seq1.2R	TTGCCCCACTAAAAGTCC
2	XL-PCR_F2	TGTCTCAGCCGGGAGCATATTTA
	XL-PCR-F3	GAAAAGGTGCCAATGGCTGATTA
	XL-PCR_R2	GCGTAATAACTGCCCGCATTACA
	mtDNA_Seq37R	CATTGGCACCTTTTCATTGT
	mtDNA_Seq38R	GGCAAGCTTTAAATGCAAAT
	mtDNA_Seq39R	TGAGAGATAATTCTGCCATTTG
	mtDNA_Seq40R	AATCTGGTTCCCAAAGAAAT
	mtDNA_Seq41R	GGTTTCTATCTCAGGGCTTT
	mtDNA_Seq42R	ATACGCTCGAGTGTCTGAGT
	mtDNA_Seq9F	AAAAGGGGCTTTGATTTCTT
	mtDNA_Seq6F	ACTGATACCCCCTTTTGACA
3	mtDNA_Seq34R	GGGGTGGAACTTTGGGTCTT
	mtDNA_Seq35R	TCCTGCTCACATTCAACCTG
	mtDNA_Seq10F	TTAGCTCACAATCCGTCTGA
	CytBF	CAAAGGTTTGCCCACCTAGTTCC
	CytBR	CCAAACTCCGCGGTTACTACAGA
4	XL-PCR-R3.4	ATGGAAAGGGTGGCGAGTTA
	Lsal-mt14R	TGCTTACTCCTCCGTAGGTC
	mtDNA_Seq61F	AGCTAAGGGAAGTCATAAAT
	mtDNA_Seq28F	GGTCTAGGGGCTAGGGTGCT
	mtDNA_Seq17F	CTAGGAGAGAGGCTGACGAG
	mtDNA_Seq21R	GAGCTCCTCAATAGCAACAA
	CT_Seq2.1F	CAGCATTGAGTAAGCAAGC
5	mtDNA_10F	TTAGCTCACAATCCGTCTGA
	mtDNA_48R	TTAGGCCCAAACTTTTCTACT
	prom2F	CCCCAATGGATTTGAGGACCCTGTTGAATG
	prom3F	TCAGAAAGATATCTGGCCCCAGGGGAGCAC
	CT_Seq3.1F	ATCCCGCATGATATGGTC
	CT_Seq3.2F	TTCCGGTCATAATTTGGG
6	mtDNA_48F	AGTAGAAAAGTTTGGGCCTAAA
	mtDNA_56R	TTGACTTTCCAAATCTTTTA
	CT_Seq1.2F	GCTACGTTCCCCAAAGG
	CT_Seq4.1F	GCAGGGTAAAATTGACGG
	CT_Seq4.2F	AGAGTTAATTGTGGCAGGG
	CT_Seq5.2F	ATTTGGAAAGTCAAGGAGG
	CT_R1	ATATTTCTCGCCGCTGGTATATTTT
	T7	TAATACGACTCACTATAGGG
	SP6	CATTTAGGTGACACTATAG

Supplementary Table S7. Association between mtDNA haplotype and DTM susceptibility status. Fisher's exact test was used to assess the association between the mtDNA haplotype assigned and the classification of lice as resistant or susceptible (4x2 table). Mitochondrial DNA haplotype and DTM resistance were significantly associated ($P = 1.746 \cdot 10^{-5}$). Pairwise comparisons between haplotypes are shown in the table.

Comparison	P-value	Adj. P-value
H1 : H2	$1.02 \cdot 10^{-5}$	$6.12 \cdot 10^{-5}$
H1 : H3	$3.61 \cdot 10^{-3}$	$7.22 \cdot 10^{-3}$
H1 : H4	$5.13 \cdot 10^{-4}$	$1.54 \cdot 10^{-3}$
H2 : H3	$2.78 \cdot 10^{-1}$	$3.41 \cdot 10^{-1}$
H2 : H4	$6.02 \cdot 10^{-1}$	$6.02 \cdot 10^{-1}$
H3 : H4	$2.84 \cdot 10^{-1}$	$3.41 \cdot 10^{-1}$

Supplementary Table S8. Mitochondrial haplotypes expressed in *L. salmonis* isolates of Scottish origin. See Supplementary Table S9 for details about the isolates studied.

Position[a]	Alleles	1A	1B	1C	1D	1E	1F	1G	1H	2	3	4
Synonymous SNPs												
3338	G/A	G	G	G	G	G	G	G	G	A	G	G
5889	T/C	T	T	T	T	T	T	T	T	C	T	C
8134	G/A	G	G	G	G	G	G	G	G	A	G	A
8600	T/C	T	T	T	T	T	T	T	T	C	C	C
Synonymous SNPs												
714	A/G	A	A	A	A	A	A	A	A	G	A	G
1174	G/A	G	G	G	G	G	G	G	G	A	G	A
1678	C/T	C	T	T	C	C	C	T	C	T	C	T
3056	C/T	C	T	T	C	C	C	T	C	T	C	T
4563	G/A	G	G	G	G	G	G	A	G	A	G	A
6325	G/A	G	G	G	G	G	G	G	G	A	G	A
9030	A/G	A	A	A	A	A	A	A	G	G	A	G
9426	A/G	A	G	G	A	G	G	A	A	G	A	G
10094	G/A	G	G	G	G	G	G	G	G	A	G	A
10722	T/TAG	T	T	T	T	T	T	T	T	TAG	T	TAG
11190	C/T	C	T	T	C	C	C	T	C	T	C	T
13466	G/A	G	A	A	A	A	G	A	G	A	G	A
14013	A/G	A	A	G	A	A	A	A	A	G	A	G
14751	C/T	C	T	T	C	C	C	T	C	T	C	T

[a] Numbering according to a *L. salmonis* mitochondrial genome assembly generated for a Scottish isolate (NCBI Accession number LT630766.1).

Supplementary Table S9. Association of mitochondrial haplotypes with deltamethrin resistance in *L. salmonis* isolates of Scottish origin. The specimens genotyped included lice collected from wild salmonids and at Scottish aquaculture farm sites, as well as individuals of *L. salmonis* strains NA01-O, IoA-00 and IoA-02 characterised previously[1].

Year of isolation	Origin	EC_{50} [µg/L] (95% CI)	N	1A	1B	1C	1D	1E	1F	1G	1H	2	3	4
2010	Wild hosts (East Lothian)	n.d.	18	55.6	-	5.6	5.6	5.6	5.6	5.6	5.6	-	-	11.1
2018	Farm site 1 (Argyll)	>2.0[a]	106	3.8	-	-	-	-	-	-	-	73.6	15.1	7.5
2018	Farm site 2 (Argyll)	>2.0[b]	54	3.7	1.9	-	-	-	-	-	-	75.9	7.4	11.1
2019	Farm site 3 (Argyll)	8.00 (5.7-10.2)[c]	28	-	-	-	-	-	-	-	-	71.4	21.4	7.1
2012	NA01-O	24.8 (12.2-85.7)[d]	20	-	-	-	-	-	-	-	-	70	10	20
2003	IoA-00	0.25 (0.2-0.3)[e]	22	68.2	31.8	-	-	-	-	-	-	-	-	-
2011	IoA-02	25.95 (18.0-33.9)[e]	24	-	-	-	-	-	-	-	-	100	-	-

[a] Single-dose bioassay: 97.2% (NTotal=106) remained unaffected after exposure to 2 µg/L deltamethrin. [b] Single-dose bioassay: 94.4% (N Total=54) remained unaffected after exposure to 2 µg/L deltamethrin[2]. [c] EC_{50} according to Tschesche et al.[2]. [d] EC_{50} according to Carmona-Antoñanzas[1]. [e] Raw data used to derive EC_{50} are provided in Supplementary Data S4.

Supplementary Table S10. Sequence variations specific to *L. salmonis* from laboratory strain IoA-10.

Position[a]	Type	Location	Description	Reference	Alternative
246	Polymorphism	Intergenic		C	T
734	Polymorphism	Intergenic		T	C
810	Deletion	Intergenic		GA	G
815	Polymorphism	Intergenic		T	C
839	Polymorphism	Intergenic		T	C
891	Insertion	Intergenic		A	AAT
895	Polymorphism	Intergenic		C	T
1175	Non-synonymous	ND4	CTC/Leu → CCC/Pro	A	G
1788	Non-synonymous	ND4	ACC/Thr → GCC/Ala	T	C
2068	Synonymous	ND4	TTG/Leu → TTA/Leu	C	T
2412	Synonymous	ND2	TTG/Leu → CTG/Leu	T	C
2643	Synonymous	ND2	CTA/Leu → TTA/Leu	C	T
3050	Synonymous	ND2	GTT/Val → GTC/Val	T	C
3540	Synonymous	COX3	TTG/Leu → TTA/Leu	G	A
4341	Synonymous	ND3	AGC/Ser → AGA/Ser	C	A
5254	Synonymous	ND5	GCT/Ala → GCC/Ala	T	C
5905	Synonymous	ND5	TCT/Ser → TCC/Ser	T	C
6122	Synonymous	ND5	CTA/Leu →TTA/Leu	C	T
6478	Polymorphism	tRNA-Met		A	G
6774	Synonymous	COX2	GGC/Gly → GGG/Gly	C	G
6870	Synonymous	COX2	GTA/Val → GTG/Val	A	G
7056	Synonymous	COX2	CGC/Arg → CGT/Arg	C	T
7398	Synonymous	ND1	CCT/Pro → CCC/Pro	T	C
7675	Synonymous	ND1	CTG/Leu → TTG/Leu	C	T
8627	Non-synonymous	COX1	GTA/Val → GCA/Ala	T	C
9619	Synonymous	COX1	TTG/Leu → CTG/Leu	T	C
10951	Polymorphism	Intergenic		A	G
12015	Synonymous	ND6	TTA/Leu → CTA/Leu	T	C
12126	Polymorphism	tRNA-Lys		A	G
12334	Synonymous	ND4L	TCC/Ser → TCT/Ser	G	A
13230	Non-synonymous	ATP6	ATT/Ile → GTT/Val	A	G
13460	Synonymous	ATP6	ACC/Thr → ACG/Thr	C	G
14502	Synonymous	CYTB	TGA/Trp → TGG/Trp	T	C
14841	Synonymous	CYTB	TCG/Ser → TCC/Ser	C	G
15384	Polymorphism	Insertion		C	T
15520	Polymorphism	Insertion		A	C

[a] Numbering according to *L. salmonis* mitochondrial reference genome (NCBI Accession number LT630766.1).

Supplementary Table S11. Mitochondrial haplotypes of *L. salmonis* first filial (F1) progenies derived from parental (P0) crosses of different sex-strain orientations. Ten individuals of each cross were subjected to PCR based genotyping assays at 18 mitochondrial SNPs, which have been described in a previous study[1].

Position	Alleles	IoA-02	IoA-10	IoA-00	IoA-00 dam × IoA-10 sire	IoA-10 dam × IoA-00 sire
Non-synonymous SNPs						
3338	G/A	A	G	G	G	G
5889	T/C	C	T	T	T	T
8134	G/A	A	G	G	G	G
8600	T/C	C	C	T	T	C
Synonymous SNPs						
714	A/G	G	A	A	A	A
1174	G/A	A	G	G	G	G
1678	C/T	T	C	C-T	T	C
3056	C/T	T	C	C-T	T	C
4563	G/A	A	G	G	G	G
6325	G/A	A	G	G	G	G
9030	A/G	G	A	A	A	A
9426	A/G	G	A	A-G	G	A
10094	G/A	A	G	G	G	G
10722	T/TAG	TAG	T	T	T	T
11190	C/T	T	C	C-T	T	C
13466	G/A	A	G	G-A	A	G
14013	A/G	G	A	A	A	A
14751	C/T	T	C	C-T	T	C
Haplotype		2	3	1A-1B[a]	1B	3

[a] 67 individuals were genotyped at SNPs C11190T, A9426G, and G6325A, and revealed of two haplotypes. Five individuals of each haplotype were genotyped at the remaining SNPs, which led to the identification of haplotypes 1A and 1B.

Supplementary Table S12. Sublethal effects of DTM and fenpyroximate on *L. salmonis*. Shown are behavioural responses of adult male salmon lice observed in the experiment described in Figure 3.

Strain	Treatment [concentration]	Number of animals	Rated "live"	Rated "moribund"	Rated "dead"	Affected	Dead
IoA-00	Solvent control	17	16	0	1	5.9%	6%
IoA-00	Fenpyroximate [100 µg/L]	12	5	6	1	58%	8%
IoA-00	DTM [2 µg/L]	18	0	16	2	100%	11%
IoA-02	Solvent control	19	19	0	0	0%	0%
IoA-02	Fenpyroximate [100 µg/L]	15	9	5	1	40%	7%
IoA-02	DTM [2 µg/L]	12	12	0	0	0%	0%
IoA-10	Solvent control	18	18	0	0	0%	0%
IoA-10	Fenpyroximate [100 µg/L]	11	2	8	1	82%	9%
IoA-10	DTM [2 µg/L]	19	19	0	0	0%	0%

Supplementary Figures

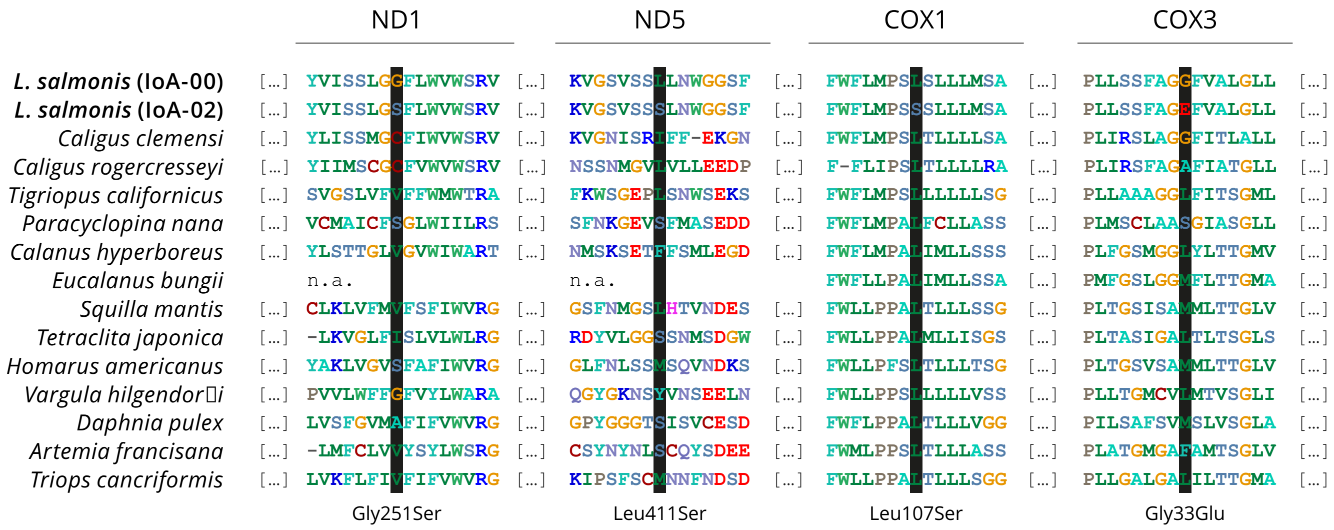

Supplementary Figure S1. Partial alignment of the predicted amino acids encoded by NADH dehydrogenase subunits 1 (ND1) and 5 (ND5), and cytochrome-c-oxidase subunits 1 (COX1) and 3 (COX3) in *L. salmonis* and 13 crustacean species. Deltamethrin resistance in *L. salmonis* strains derived from fioeld isolates has previously been shown to be associated with four non-synonymous single nucleotide polymorphisms in the mitochondrial genome, G3338A (COX3 Gly33Glu), T5889C (ND5 Leu411Ser), G8134A (ND1 Gly251Ser), and T8600C (COX1 Leu107Ser). Positions of the amino acids affected by these SNPs are boxed in the alignment. N.a.: sequence not available. NCBI accession number: *L. salmonis* (COX3: SFW10606.1; ND5: SFW10608.1; COX1: SFW10611.1; ND1: SFW10610.1), *C. clemensis* (COX3: HQ157566.1 translation gene 3165-3998; ND5: HQ157566.1 translation gene 4583-6256; COX1: ADM67904.1; ND1: ADM67903.1), *C. rogercresseyi* (COX3: HQ157565.1 translation gene 3177-3995; ND5: ADM67895.1; COX1: HQ157565.1 translation gene 8213-9751; ND1: ADM67897.1), T. californicus (COX3: ABI33097.1; ND5: ABI33098.1; COX1: ABI33091.1; ND1: ABI33093.1), *P. nana* (COX3: ACK86653.1; ND5: ACK86649.1; COX1: ACK86645.1; ND1: ACK86655.1), *C. hyperboreus* (COX3: YP_007026102.1; ND5: YP_007026108.1; COX1: YP_007026098.1; ND1: YP_007026109.1), *E. bungii* (COX3: BAD19000.1; COX1: BAD18993.1), *S. mantis* (COX3: YP_054549.1; ND5: YP_054551.1; COX1: YP_054545.2; ND1: YP_054556.2), *T. japonica* (COX3: YP_022492.1; ND5: YP_022494.1; COX1: YP_022488.1; ND1: YP_022499.1), *H. americanus* (COX3: YP_004563975.1; ND5: YP_004563977.1; COX1: YP_004563971.1; ND1: YP_004563982.1), *V. hilgendorfii* (COX3: NP_954731.1; ND5: NP_954732.1; COX1: NP_954727.1; ND1: NP_954739.1), *D. pulex* (COX3: NP_008626.1; ND5: NP_008628.1; COX1: NP_008622.1; ND1: NP_008633.1), *A. franciscana* (COX3: NP_007113.1; ND5: NP_007115.1; COX1: NP_007109.1; ND1: NP_007120.1), *T. cancriformis* (COX3: NP_775070.1; ND5: NP_775072.1; COX1: NP_775066.1; ND1: NP_775077

Supplementary Data

Supplementary Data S1

Raw data of deltamethrin bioassays with copepodid *L. salmonis* larvae (Table 3). [CSV]

Supplementary Data S2

MtDNA SNP genotypes in historical *L. salmonis* samples. Data from Tjensvoll et al.[3], who have reported sequences of four mtDNA genes in a total of 180 lice from six Atlantic populations, were re-analysed to derive genotypes for eight mtDNA SNP loci studied in this report and assign samples to mtDNA haplotypes. For each SNP locus, “0” denotes the allele found in haplotype 1A, while “1” indicates the alternative allele (see Supplementary Table S8). [CSV]

Supplementary Data S3

MtDNA SNP genotypes in DTM resistant and susceptible *L. salmonis* strains derived from Norwegian isolates. Bakke et al.[4] have reported mtDNA SNPs in DTM susceptible (strain S) and resistant (strain R) *L. salmonis* strains. The SNP loci assessed

include 16 SNPs studied in this report, for which genotypes expressed in the Norwegian strains have been compiled. For each SNP locus, “0” denotes the allele found in haplotype 1A, while “1” indicates the alternative allele (see Supplementary Table S8). [CSV]

Supplementary Data S4

Raw data of deltamethrin bioassays with preadult/adult *L. salmonis* (Figure 2). [CSV]

Supplementary Data S5

Raw data of ATP measurements in male adult *L. salmonis* following drug exposures (Figure 3). [CSV]

References

1. Carmona-Antoñanzas, G. *et al.* Maternal inheritance of deltamethrin resistance in the salmon louse *Lepeophtheirus salmonis* (Krøyer) is associated with unique mtDNA haplotypes. *PloS One* **12**, e0180625, DOI: 10.1371/journal.pone.0180625 (2017).
2. Tschesche, C. *et al.* Investigation of deltamethrin resistance in salmon lice (*Lepeophtheirus salmonis*) provides no evidence for roles of mutations in voltage-gated sodium channels. *Pest Manag. Sci.* **77**, 1052–1060, DOI: 10.1002/ps.6120 (2021).
3. Tjensvoll, K., Glover, K. A. & Nylund, A. Sequence variation in four mitochondrial genes of the salmon louse *Lepeophtheirus salmonis*. *Dis Aquat Organ* **68**, 251–259, DOI: 10.3354/dao068251 (2006).
4. Bakke, M. J., Agusti, C., Bruusgaard, J. C., Sundaram, A. Y. M. & Horsberg, T. E. Deltamethrin resistance in the salmon louse, *Lepeophtheirus salmonis* (Krøyer): Maternal inheritance and reduced apoptosis. *Sci Rep* **8**, 8450, DOI: 10.1038/s41598-018-26420-6 (2018).
